# A complementary low-Schottky-barrier S/D-based nanoscale dopingless bidirectional reconfigurable field effect transistor with an improved forward current

**DOI:** 10.1186/s11671-023-03835-3

**Published:** 2023-03-24

**Authors:** Xiaoshi Jin, Shouqiang Zhang, Chunrong Zhao, Meng Li, Xi Liu

**Affiliations:** School of Information Science and Engineering, Shenyang University of Technology, Shenyang, 110870 China

**Keywords:** Schottky barrier height, Reconfigurable, Forward current, Field effect transistor

## Abstract

In this paper, a nanoscale dopingless bidirectional RFET (BRFET) is proposed. Unlike conventional BRFETs, the proposed BRFET uses two different metal materials to form two different types of Schottky barriers on the interface between the S/D and silicon. For one of the two metal forms, the Schottky barrier height between the conduction band of the semiconductor and one of the two metal materials is lower than half of the energy band gap. The Schottky barrier height between the valence band of the semiconductor and the other kind of the two metal materials is lower than half of the energy band gap of the semiconductor. Therefore, a complementary low Schottky barrier (CLSB) is formed. Therefore, more carriers from the source electrode can easily flow into the semiconductor region through thermionic emission in both n-mode and p-mode compared to conventional BRFET operation, which generates carriers through the band-to-band tunneling effect. Therefore, a larger forward current can be achieved by the proposed CLSB-BRFET. The performance of the CLSB-BRFET is investigated by device simulation and compared with that of the BRFET. The working principle is interpreted through an analysis based on energy band theory. The output characteristics and reconfigurable function are also investigated and verified.

## Introduction

Today, the minimum size of planar complementary metal oxide semiconductor (CMOS) integrated technology is about to reach the physical limit. Therefore, when the physical density of the circuit can no longer depend on the primary strategy of reducing the device size, it is necessary to find new, secondary ways to improve the functional density of the circuit. This means that the designers should use fewer devices to achieve richer functions through circuit design or develop new devices with richer functions through device design to reduce the dependence on the number of devices. Related research has become a hot issue in recent years. The reconfigurable field effect transistor (RFET) and bidirectional RFET (BRFET) have been proposed. As a single field effect transistor, the conduction mode can be configured as n-type or p-type by resetting the voltage applied on the program gate (PG) during operation [1, 2]. The RFET offers an advantage in logic arrays and enables various logic gates with fewer transistors than metal oxide semiconductor field effect transistor (MOSFET)-based CMOS technology [3–7]. To realize the reconfigurable function, the RFET needs to form a high Schottky barrier on the source side between the metal source and the conduction band of the semiconductor and the valence band of the semiconductor at the same time. Thereafter, nickel silicide (NiSi) is usually adopted to form a Schottky barrier in RFETs. The Schottky barrier height formed between NiSi and the semiconductor conduction band qϕ_bn0_ is similar to that formed between NiSi and the semiconductor valence band qϕ_bp0_ [8]. This is appropriate for reconfiguration operation. The PG is used to generate a strong field effect on the source side to overcome the Schottky barrier, which prevents the carriers flowing from the source electrode into the conduction band or valence band of the semiconductor region. Through the strong field effect, band bending is generated in the semiconductor near the source side, and the band-to-band tunneling (BTBT) effect occurs and generates electron–hole pairs. If the voltage applied on the PG (V_PG_) is positive, electrons accumulate in the semiconductor region near the source electrode, and the device works in n-mode, while if V_PG_ is negative, holes accumulate, and the device works in p-mode. Therefore, the magnitude of the PG voltage determines the intensity of band bending and the corresponding number of carriers generated by BTBT, and the polarity of the PG voltage determines the type of carriers that the source can provide and determines the conduction type of the RFET. However, because the number of carriers generated by the tunneling effect is limited, the forward conduction current of the conventional BRFET is much smaller than that of the MOSFET, which is a fundamental disadvantage of the unique Schottky barrier-based RFET. Moreover, the Schottky barrier MOSFET (SB-MOSFET) is also a type of device that forms metallic source/drain (S/D) junctions instead of the p–n junctions formed in the MOSFET [9–11]. In addition, the metallic S/D architecture holds the advantage of relaxing the severe constraints imposed on the conventional implanted S/D of MOSFETs. Taking p-type SB MOSFETs as an example, the height of the Schottky barrier for holes in the valence band ϕ_bp_ is much smaller than that for electrons in the conduction band ϕ_bn_. Therefore, unlike the RFET, the SB-MOSFET uses metal materials with smaller ϕ_bn_ (n-type) or ϕ_bp_ (p-type) to reduce the height of the Schottky barrier as much as possible. This makes it unnecessary for carriers to flow into semiconductors through the BTBT effect, but they can directly flow into silicon in massive quantities through thermionic emissions. The forward current can be largely enhanced by reducing the height of the Schottky barrier. However, like the RFET, the SB-MOSFET is also a device with a unique metal silicide S/D based on a hologenetic S/D Schottky barrier. Because the sum of qϕ_bn_ and qϕ_bp_ equals the energy band gap of the semiconductor, if a material achieves a lower qϕ_bn_, then qϕ_bp_ is large. Therefore, for example, even if a conventional BRFET adopts a low qϕ_bn_ material to form the source electrode, only the forward current in n-mode can be effectively enhanced, and the carriers that are supplied in p-mode still depend on BTBT. Based on the above analysis, we found that if we use a material that forms a high Schottky barrier for both the conduction band and the valence band, the forward conduction current is low in both p-mode and n-mode. If we use a material that forms a low Schottky barrier for either the conduction band or for the valence band, the forward current can be improved only in one mode, while the forward current cannot readily be improved in the other mode. Therefore, to improve the forward current of the BRFET in both modes, in this paper, we propose a complementary low-Schottky-barrier S/D-based nanoscale dopingless bidirectional RFET (CLSB-BRFET). Unlike devices that adopt only one type of metal material to form the S/D electrodes, such as the conventional BRFET or SB MOSFET, the proposed CLSB-BRFET utilizes two different metal materials to form two different types of Schottky barriers on the interface between the S/D and silicon. This means that both a low ϕ_bn_ and a low ϕ_bp_ Schottky barrier can be achieved simultaneously. A low ϕ_bn_ Schottky barrier can be formed on the interface between ErSi and silicon with ϕ_bn_ ~ 0.25 V [12], and a low ϕ_bp_ Schottky barrier can be formed on the interface between platinum silicide (PtSi) and silicon with ϕ_bp_ ~ 0.25 V [13, 14]. Therefore, when the PG is exposed to a positive voltage, the material that forms the lower ϕ_bn_ becomes dominant. The electrons from the source electrode can easily flow into the semiconductor region mainly through the lower-ϕ_bn_ Schottky barrier due to thermionic emission but not through the other material, which forms a higher ϕ_bn_ due to BTBT. When the PG is exposed to a negative voltage, the holes from the semiconductor can easily flow into the source electrode through the lower-ϕ_bp_ Schottky barrier due to thermionic emission but not through the other material, which forms a higher ϕ_bp_ due to BTBT. In this work, the characteristics of the proposed CLSB-BRFET are compared with those of the conventional BRFET, and the working principle is interpreted through an analysis based on energy band theory. Compared to the conventional BRFET, the proposed CLSB-BRFET can achieve a much higher on-state current and much lower static power consumption.

## Methods

**Fig. 1:**
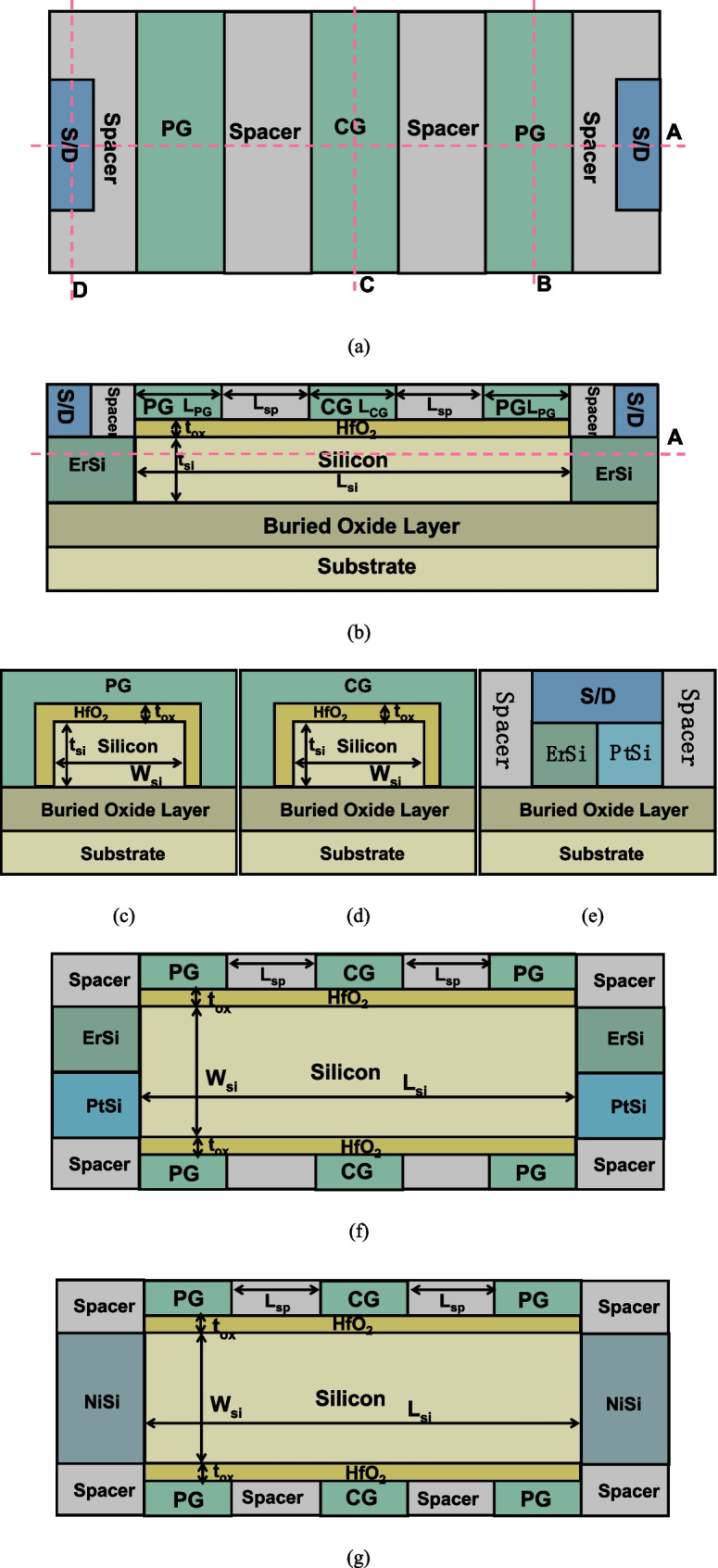
**a** The top view of the proposed CLSB-BRFET, **b** the cross-sectional views along cut line A of **a**, **c** the cross-sectional views along cut line B of **a**, **d** the cross-sectional views along cut line C of **a**, **e** the cross-sectional views along cut line D of **a**, **f** the cross-sectional view along cut line A of **b**, and **g** a cross-sectional view of a conventional unique Schottky barrier-based BRFET

Figure 1a shows the top view of the proposed CLSB-BRFET, and Fig. 1b–e show the cross-sectional views along cut line A, cut line B, cut line C, and cut line D of Fig. 1a, respectively. Figure 1f shows the cross-sectional view along cut line A of Fig. 1b. Figure 1g shows a cross-sectional view of a conventional unique Schottky-barrier-based BRFET. L_si_ is the length of the silicon, L_CG_ is the length of the CG, L_PG_ is the length of the PG on either the source or side, L_sp_ is the length of the spacer between the CG and the PG, t_si_ is the thickness of silicon, t_ox_ is the HfO_2_ gate oxide thickness, W_si_ is the width of the silicon, ε_HfO2_ is the relative permittivity of HfO_2_, ε_spacer_ is the relative permittivity of the spacer, qϕ_bn1_ is the barrier height between the first kind of metal, erbium silicide (ErSi), of the S/D electrodes and the conduction band of silicon, qϕ_bp1_ is the barrier height between the first kind of metal (ErSi) of the S/D electrodes and the valence band of the silicon, qϕ_bn2_ is the barrier height between the second kind of metal (PtSi) of the S/D electrodes and the conduction band of the silicon, and qϕ_bp2_ is the barrier height between the second kind of metal (PtSi) of the S/D electrodes and the valence band of the silicon. The parameter selection for the CLSB-BRFET is shown in Table 1. The parameters of the conventional BRFET are selected to be as consistent as possible with the parameters of the CLSB-BRFET. The device performances are verified through SILVACO Atlas tools [15]. The Atlas device simulation tool consists of a set of fundamental equations that link the electrostatic potential and the carrier densities. These equations, which are solved inside any general-purpose device simulator, have been derived from Maxwell’s laws and consist of Poisson’s equation, the continuity equations, and the drift–diffusion transport equations. Poisson’s equation relates variations in electrostatic potential to local charge densities. The continuity and transport equations describe the way that the electron and hole densities evolve as a result of transport processes, generation processes, and recombination processes. The drift–diffusion transport model, Fermi–Dirac statistic model, Auger recombination model, FN tunnel model and BTBT model are all turned on in all simulation work. There are two kinds of Schottky barriers formed between the S/D electrodes and the semiconductor (silicon). We take an n-mode operation as an example. Because qϕ_bn1_ is much smaller than qϕ_bp1_, the Schottky barrier between ErSi and the conduction band of the silicon is lower than the Schottky barrier between ErSi and the valence band of the silicon. Therefore, when the PG is positively biased, the electrons from the source metal material, ErSi, more easily flow into the conduction band of the silicon through the thermionic emission effect compared to operation of the conventional unique Schottky barrier-based BRFET.

**Table 1:** Parameter selection for the CLSB-BRFET and BRFET

Parameters	Values/Units
The length of the semiconductor (silicon) (L_si_);	30 nm
The length of the control gate (L_CG_);	6 nm
The length of the program gate (L_PG_);	6 nm
The length of the spacer (L_sp_)	6 nm
The HfO_2_ oxide layer thickness (t_ox_);	1 nm
The thickness of the semiconductor (silicon) (t_si_);	5 nm
The width of the semiconductor (silicon) (W_si_);	5 nm
The relative permittivity of HfO_2_ (ε_HfO2_);	21.976
The relative permittivity of the spacer (ε_spacer_);	3.89
The barrier height between the ErSi S/D electrodes and the conduction band of the silicon (qϕ_bn1_);	0.25 eV
The barrier height between the ErSi S/D electrodes and the valence band of the silicon (qϕ_bp1_);	0.83 eV
The barrier height between the PtSi S/D electrodes and the conduction band of the silicon (qϕ_bn2_);	0.83 eV
The barrier height between the PtSi S/D electrodes and the valence band of the silicon (qϕ_bp2_);	0.25 eV
The barrier height between the NiSi S/D electrodes and the conduction band of the silicon (qϕ_bn0_);	0.56 eV
The barrier height between the NiSi S/D electrodes and the valence band of the silicon (qϕ_bp0_);	0.52 eV
The drain to source voltage (V_DS_)	− 0.6 V–0.6 V
The control gate to source voltage (V_GS_)	− 0.6 V–0.6 V
The program gate voltage (V_PG_)	− 0.6 V–0.6 V
The Fermi level of the source electrode (E_FMS_)	0 eV
The Fermi level of the source electrode (E_FMD_)	− 0.4 eV
The electron concentration (n)	cm^−3^
The surface electron concentration (n_s_)	cm^−3^
The hole concentration (p)	cm^−3^
The surface hole concentration (p_s_)	cm^−3^
The electron quasi-Fermi level (E_FN_)	eV
The hole quasi-Fermi level (E_FP_)	eV
The effective density of states of the conduction band (N_C_)	cm^−3^
The effective density of states of the conduction band (N_V_)	cm^−3^
The band energy of the bottom of the conduction band (E_C_)	eV
The band energy of the top of the valence band (E_V_)	eV
The threshold voltage (V_TH_)	V
The on-state and off-state current ratio (I_on_/I_off_)	
The subthreshold swing (SS)	mV/decade

## Results and discussion

**Fig. 2:**
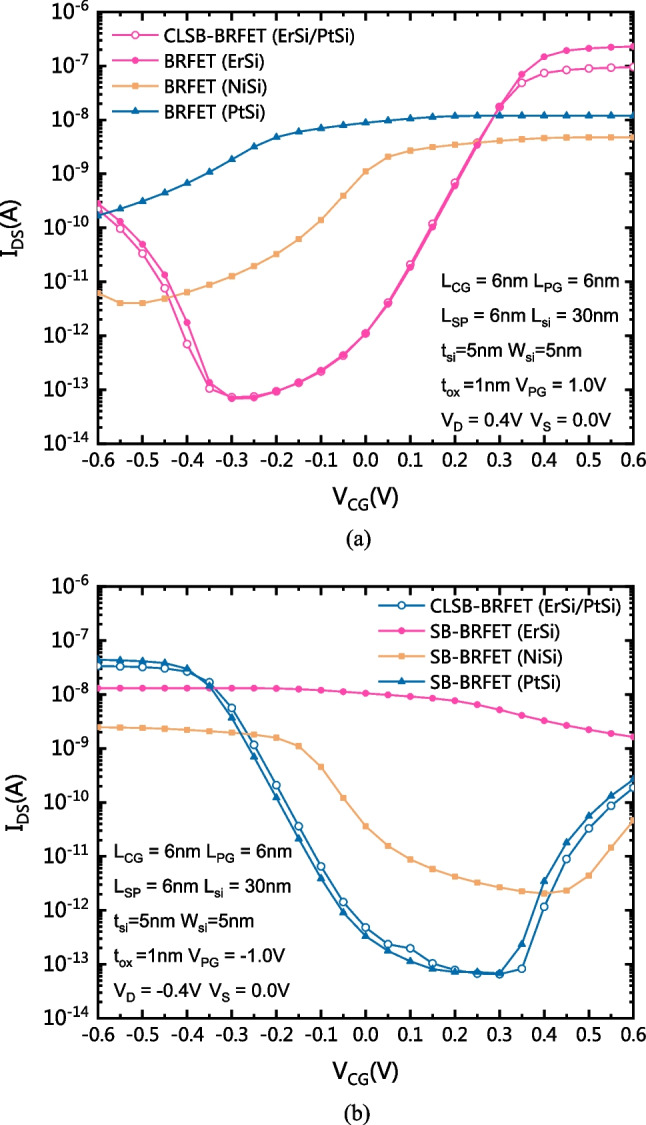
**a** Comparison of transfer characteristics between the CLSB-BRFET and conventional BRFETs with different S/D materials in n-mode. **b** Comparison of transfer characteristics between the CLSB-BRFET and conventional BRFETs with different S/D materials in p-mode

Figure 2a shows a comparison of the transfer characteristics between the CLSB-BRFET and the conventional BRFET with different S/D metal materials in n-mode. Figure 2b shows the comparison of transfer characteristics between the CLSB-BRFET and the conventional BRFET with different S/D metal materials in p-mode. It is obvious that the CLSB-BRFET is the only device of the group that can achieve a higher forward conduction current and better subthreshold characteristics in both n-mode and p-mode at the same time. As Fig. 2a shows, for the conventional BRFET with the ErSi S/D electrode, the Schottky barrier height between the S/D electrode and the conduction band of the semiconductor is smaller than in the other cases. Therefore, the forward current of the BRFET with ErSi S/D electrodes in n-mode is the largest. Because the CLSB-BRFET uses both ErSi and PtSi as the S/D metal materials, when the device works in n-mode, the forward current generated by thermionic emission through the ErSi in the conduction band is much larger than that generated by BTBT. In this case, the role of the ErSi becomes dominant. Similarly, as Fig. 2b shows, for the conventional BRFET with the PtSi S/D electrode, the Schottky barrier height between the S/D electrode and the valence band of the semiconductor is smaller than in the other cases. Therefore, the forward current of the BRFET with PtSi S/D electrodes in p-mode is the largest. For the CLSB-BRFET in p-mode, the forward current generated by thermionic emission through the PtSi in the valence band is also much larger than that generated by BTBT. In this case, the role of the PtSi is dominant.

**Fig. 3:**
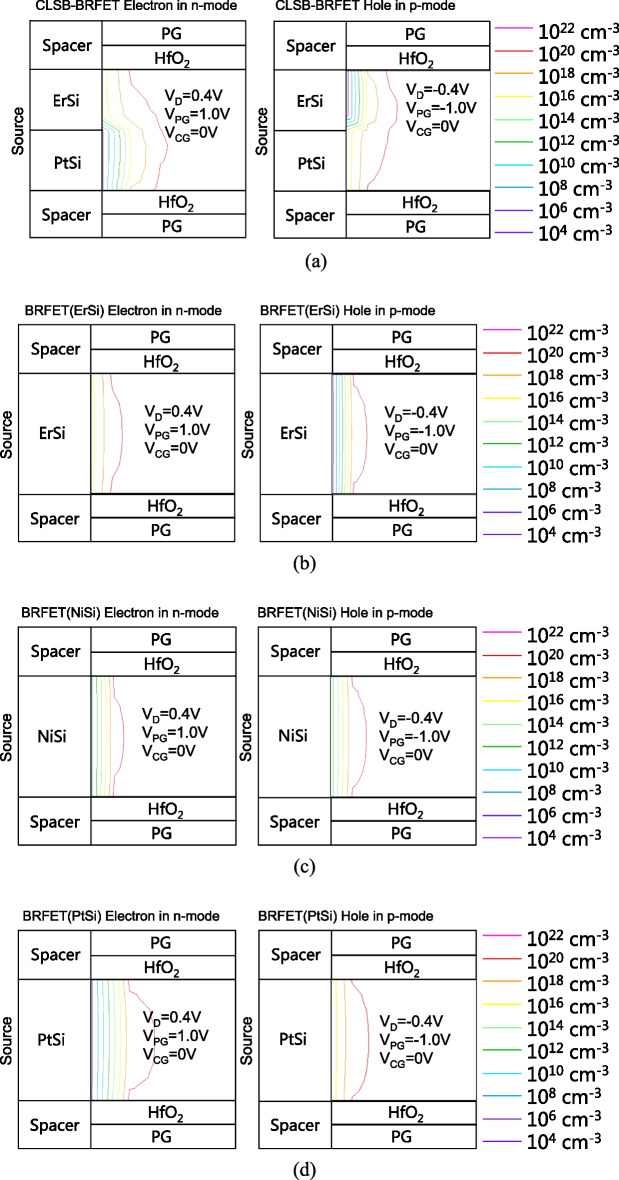
**a** The electron concentration of the CLSB-BRFET near the source side in n-mode and hole concentration of the CLSB-BRFET near the source side in p-mode. **b** The electron concentration of the BRFET with ErSi S/D electrodes near the source side in n-mode and the hole concentration of the BRFET with ErSi S/D electrodes near the source side in p-mode. **c** The electron concentration of the BRFET with NiSi S/D electrodes near the source side in n-mode and the hole concentration of the BRFET with NiSi S/D electrodes near the source side in p-mode. **d** The electron concentration of the BRFET with PtSi S/D electrodes near the source side in n-mode and the hole concentration of the BRFET with PtSi S/D electrodes near the source side in p-mode

Figure 3a shows the electron concentration of the CLSB-BRFET near the source side in n-mode and the hole concentration of the CLSB-BRFET near the source side in p-mode. Figure 3b shows the electron concentration of the BRFET with ErSi S/D electrodes near the source side in n-mode and the hole concentration of the BRFET with ErSi S/D electrodes near the source side in p-mode. Figure 3c shows the electron concentration of the BRFET with NiSi S/D electrodes near the source side in n-mode and the hole concentration of the BRFET with NiSi S/D electrodes near the source side in p-mode. Figure 3d shows the electron concentration of the BRFET with PtSi S/D electrodes near the source side in n-mode and the hole concentration of the BRFET with PtSi S/D electrodes near the source side in p-mode. For the CLSB-BRFET, when the PG is positively biased, it works in n-mode, and the electrons flow into the semiconductor region through the ErSi part of the source electrode. The electron concentration can be expressed as

1 $$n = N_{C} \exp \left( {\frac{{E_{FN}^{{}} - E_{C} }}{kT}} \right)$$

On the surface between the silicon and source electrode, the band energy difference between E_FN_ and E_C_ equals − qϕ_bn_. Therefore, the surface electron concentration can be expressed as

2 $$n_{s} = N_{C} \exp \left( {\frac{{ - q\varphi_{bn} }}{kT}} \right)$$

For ErSi, qϕ_bn1_ is approximately 0.25 V, which is the smallest among ErSi, NiSi and PtSi; therefore, the surface electron concentration of ErSi is the largest. That is approximately 10^15^ cm^−3^; for NiSi, n_s_ is approximately 10^11^ cm^−3^, and for PtSi, n_s_ is approximately 10^6^ cm^−3^. Similarly, the hole concentration can be expressed as

3 $$p = N_{V} \exp \left( {\frac{{E_{V} - E_{FP}^{{}} }}{kT}} \right)$$

On the surface between the silicon and source electrode, the band energy difference between E_FP_ and E_V_ equals − qϕ_bp_. Therefore, the surface hole concentration can be expressed as

4 $$p_{s} = N_{V} \exp \left( {\frac{{ - q\varphi_{bp} }}{kT}} \right)$$

For ErSi, qϕ_bn1_ is approximately 0.83 V, the largest value among ErSi, NiSi and PtSi; therefore, the surface hole concentration of ErSi is the smallest. That is approximately 10^6^ cm^−3^; for NiSi, p_s_ is approximately 10^11^ cm^−3^, and for PtSi, p_s_ is approximately 10^15^ cm^−3^.

**Fig. 4:**
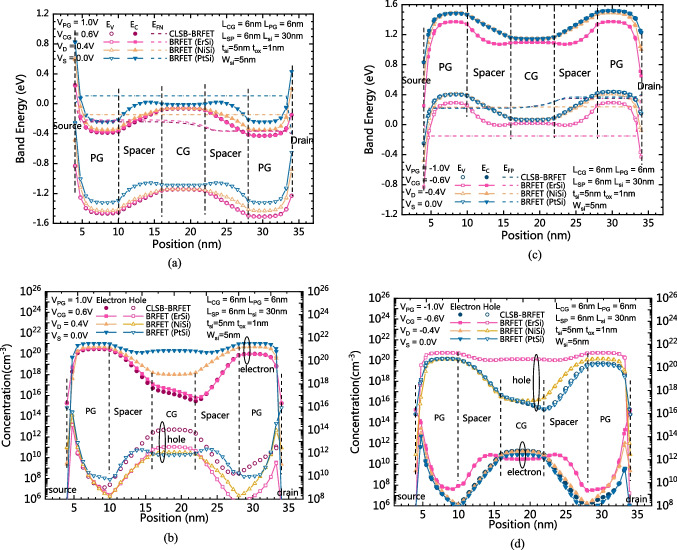
**a** The comparison of the band energy distribution between the CLSB-BRFET and BRFETs with different S/D materials in the forward state of the n-mode. **b** The comparison of the carrier concentration distribution between the CLSB-BRFET and BRFETs with different S/D materials in the forward state of the n-mode. **c** The comparison of the band energy distribution between the CLSB-BRFET and BRFETs with different S/D materials in the forward state of the p-mode. **d** The comparison of the carrier concentration distribution between the CLSB-BRFET and BRFETs with different S/D materials in the forward state of the p-mode

Figure 4a and b show the comparison of band energy distributions and carrier concentration distributions between the CLSB-BRFET and the conventional BRFET in the forward biased state in n-mode, respectively. As Fig. 4a shows, the band energy is pulled down under the joint action of both the PG and the CG for all devices. However, because the Schottky barrier heights between the source and silicon are different, because electrons are the majority carrier, the surface electron concentrations of these devices are also different. Therefore, the source contact resistances are also different. For the proposed CLSB-BRFET in n-mode, the source contact resistance and the electron concentration distribution are dominated by the ErSi. Therefore, as Fig. 4b shows, the electron distribution of the CLSB-BRFET is also similar to that of the conventional BRFET with ErSi S/D electrodes. Note that even though the lowest electron concentrations of the BRFET with NiSi or PtSi S/D electrodes are larger than those of the BRFET with ErSi S/D electrodes, the forward on-state current of the BRFET with NiSi or PtSi S/D electrodes is still smaller than that of the BRFET with ErSi S/D electrodes. When the lowest electron concentration of the BRFET exceeds the surface electron concentration n_s_, the resistance generated by the Schottky contact becomes dominant, and the forward current of the device cannot be further promoted by increasing V_G_. This is also the reason that we use the material with the smallest possible qϕ_bn_ in n-mode. Figure 4c and d show a comparison of the band energy distributions and carrier concentration distributions between the CLSB-BRFET and the conventional BRFET in the forward biased state in p-mode, respectively. As Fig. 4c shows, the band energy is pulled up under the joint action of both the PG and the CG for all devices. Because holes are the majority carrier, the surface hole concentrations of these devices are also different. Therefore, the source contact resistances in p-mode are also different. For the proposed CLSB-BRFET in p-mode, the source contact resistance and the hole concentration distribution are dominated by the PtSi. Therefore, as Fig. 4d shows, the hole distribution of the CLSB-BRFET is also similar to that of the conventional BRFET with PtSi S/D electrodes. Similar to the n-mode, when the smallest hole concentration of the BRFET exceeds the surface hole concentration n_s_, the resistance generated by the Schottky contact becomes dominant, and the forward current of the device cannot be further promoted by decreasing V_G_. The material with the smallest possible qϕ_bp_ should be adopted in p-mode.

**Fig. 5:**
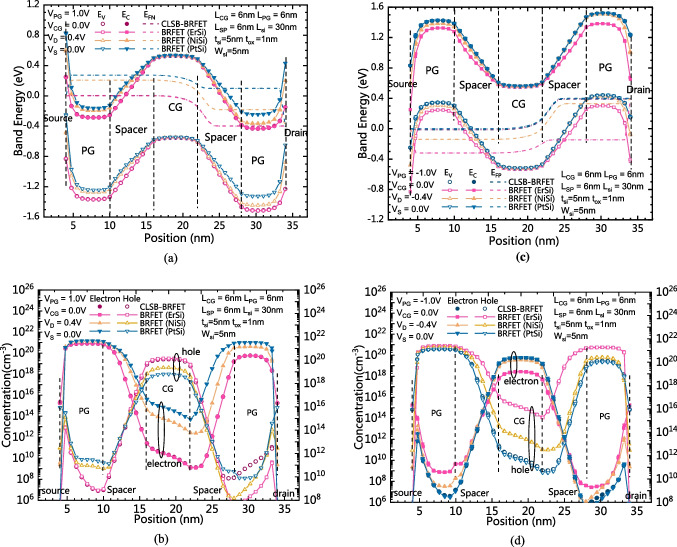
**a** The comparison of the band energy distribution between the CLSB-BRFET and the conventional BRFETs in the static state of the n-mode. **b** The carrier concentration distributions between the CLSB-BRFET and the conventional BRFETs in the static state of the n-mode. **c** The comparison of band energy distribution between the CLSB-BRFET and the conventional BRFETs in the static state of the p-mode. **d** The carrier concentration distributions between the CLSB-BRFET and the conventional BRFETs in the static state of the p-mode

Figure 5a and b show the band energy distributions and carrier concentration distributions of both the CLSB-BRFET and the conventional BRFET in the static state of the n-mode, respectively. E_FN_ of the BRFET with PtSi in the central part is the highest among all devices. E_C_ in the central part of all devices is almost the same. Therefore, according to Formula (1), as Fig. 5b shows, the electron concentration in the central part of the BRFET with PtSi S/D electrodes is the highest among these devices. Therefore, the static leakage current of the BRFET with PtSi S/D electrodes is the largest in n-mode. Because the ErSi is dominant for the proposed CLSB-BRFET in n-mode, as Fig. 2a shows, the static and subthreshold currents of the CLSB-BRFET are similar to those of the BRFET with ErSi S/D electrodes. Figure 5c and d show the band energy distribution and carrier concentration distributions of both the CLSB-BRFET and the conventional BRFET in the static state of the p-mode, respectively. E_FP_ of the BRFET with ErSi in the central part is the lowest among all devices. E_V_ in the central part of all devices is almost the same. Therefore, according to Formula (3), as Fig. 5d shows, the hole concentration in the central part of the BRFET with ErSi S/D electrodes is the highest among these devices. Therefore, the static leakage current of the BRFET with ErSi S/D electrodes is the largest in p-mode. Because PtSi is dominant for the proposed CLSB-BRFET in p-mode, as Fig. 2b shows, the static and subthreshold currents of the CLSB-BRFET are similar to those of the BRFET with PtSi S/D electrodes in p-mode.

**Fig. 6:**
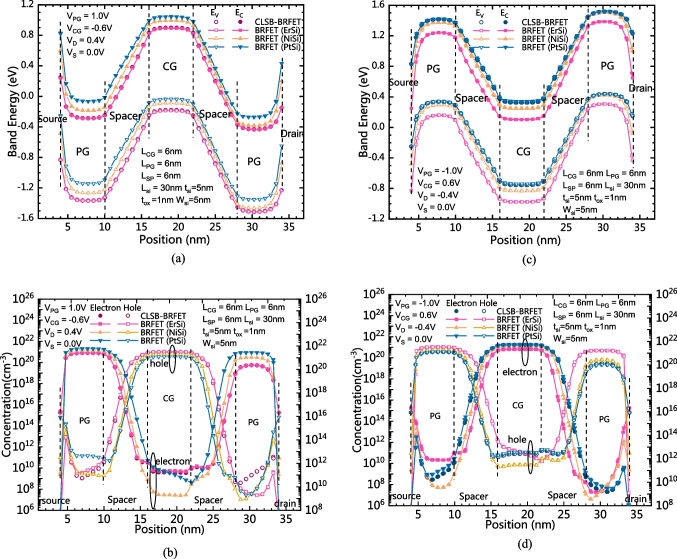
**a** The comparison of band energy distributions between the CLSB-BRFET and the conventional BRFETs in the reverse cutoff state of the n-mode. **b** The carrier concentration distributions between the CLSB-BRFET and the conventional BRFETs in the reverse cutoff state of the n-mode. **c** The comparison of band energy distributions between the CLSB-BRFET and the conventional BRFETs in the reverse cutoff state of the p-mode. **d** The carrier concentration distributions between the CLSB-BRFET and the conventional BRFETs in the reverse cutoff state of the p-mode

Figure 6a and b show a comparison of band energy distributions and carrier concentration distributions between the CLSB-BRFET and the conventional BRFETs in the reverse cutoff state of the n-mode, respectively. When the PG is positively biased and the CG is negatively biased, for both the CLSB-BRFET and the BRFETs, electrons are gathered in the semiconductor regions under the PG, while holes are gathered in the semiconductor region under the CG. As Fig. 6b shows, the semiconductor near the S/D sides is N-type, while the semiconductor at the central region is P-type. The p-type semiconductor in the central region and the n-type semiconductor near the drain side form a reversely biased PN junction when V_D_ is positive. Therefore, all devices are in the reverse cutoff state. As Fig. 6a shows, there is strong energy band bending between the CG and PG, and the energy band bending degree of all devices is almost equal. Currently, BTBT is the dominant mechanism for generating reverse leakage current. However, as Fig. 2a shows, there are some differences in the leakage current generated by different devices. The difference in S/D contact mainly causes these differences. For the CLSB-BRFET and BRFET (ErSi), as Fig. 6a shows, the built-in potential generated in the semiconductor near the drain side is much smaller than that generated by BRFET (NiSi) and BRFET (PtSi). Therefore, the electrons produced by the BTBT effect caused by the energy band bending between the CG and PG are more likely to flow out from the drain electrode. For BRFET (NiSi), the built-in potential generated is smaller than that generated by the CLSB-BRFET and BRFET (ErSi), so the electrons do not easily flow out of the drain electrode under the effect of the built-in potential. For BRFET (PtSi), although its built-in potential is the largest, the energy band bending at the interface between the semiconductor and drain electrode is also the largest. Therefore, the strongest BTBT phenomenon occurs at this interface, so the leakage current generated by BRFET (PtSi) is also greater than that generated by BRFET (NiSi). However, from another point of view, for BRFET (PtSi) and BRFET (NiSi), the potential difference between the central semiconductor region under the CG and the semiconductor region on the drain side is smaller than that of the CLSB-BRFET. Therefore, the reverse bias degree of different devices is also not the same. For BRFET (NiSi) and BRFET (PtSi), the potential differences between the center and the drain side are smaller than those of BRFET (ErSi) and the CLSB-BRFET. Therefore, compared with BRFET (ErSi) and the CLSB-BRFET, when V_CG_ equals − 0.6 V, for BRFET (NiSi) and BRFET (PtSi), their subthreshold states have just ended, while the reverse cutoff states have just begun, while for BRFET (ErSi) and the CLSB-BRFET, their subthreshold state ends earlier. In other words, BRFET (ErSi) and the CLSB-BRFET are in a deeper reverse bias state. Figure 6c shows a comparison of band energy distributions between the CLSB-BRFET and the conventional BRFETs in the reverse cutoff state of the p-mode. Figure 6d shows the carrier concentration distributions between the CLSB-BRFET and the conventional BRFET in the reverse cutoff state of the p-mode. For the p-mode, the situation of BRFET (ErSi) and BRFET (PtSi) is reversed. Similar to the forward and static states, the reverse leakage of the CLSB is also equivalent to that of BRFET(PtSi) in p-mode. Due to the high similarity between n-mode and p-mode, only the voltage applied by each electrode is reversed, so the analysis is not repeated here.

**Fig. 7:**
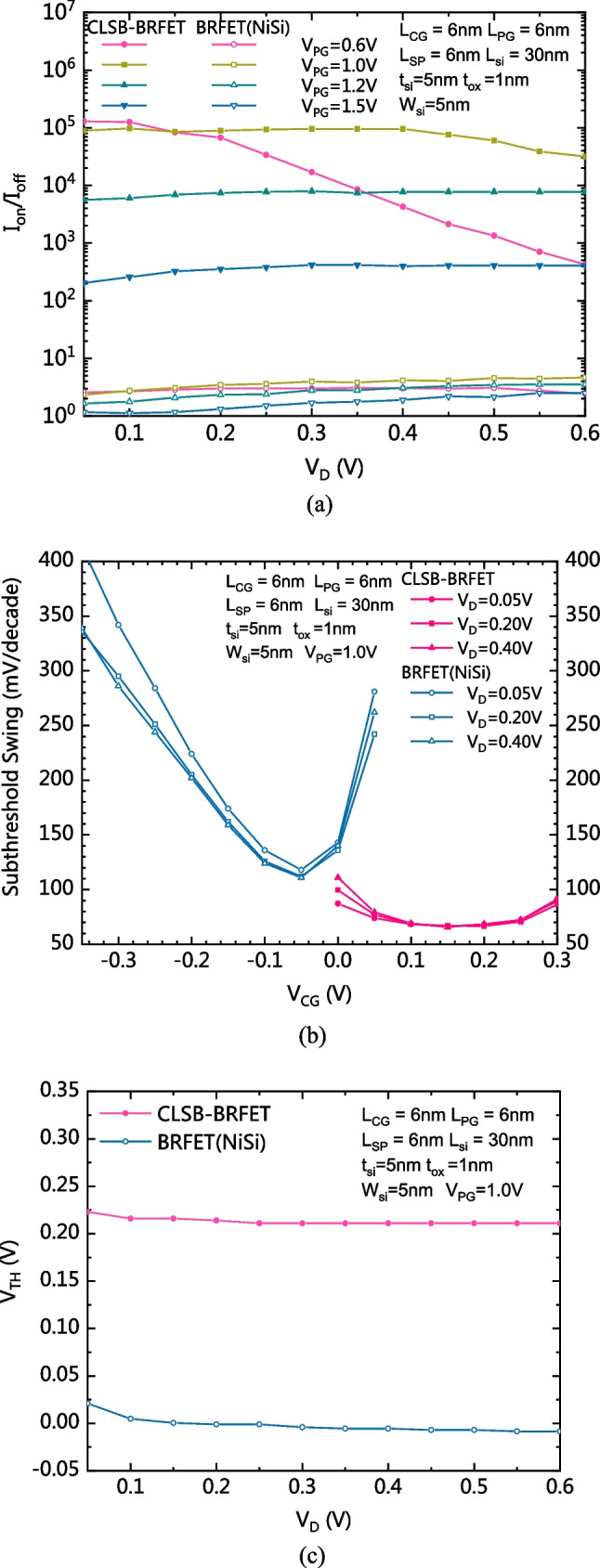
**a** Comparison of the I_on_/I_off_ current ratio between the proposed CLSB-BRFET and the conventional BRFETs. **b** Comparison of the subthreshold swing between the proposed CLSB-BRFET and the conventional BRFETs. **c** Comparison of the threshold voltage between the proposed CLSB-BRFET and the conventional BRFET (NiSi)

Figure 7a–c compare the I_on_/I_off_ current ratio, the subthreshold swing (SS) and the threshold voltage (V_TH_) between the CLSB-BRFET and the conventional BRFETs, respectively. As Fig. 7a shows, if V_PG_ is appropriately selected and large enough, V_D_ does not affect the I_on_/I_off_ ratio. The I_on_/I_off_ ratio of the CLSB-BRFET is much larger than that of the conventional BRFET with NiSi S/D electrodes. As Fig. 7b shows, if V_D_ is increased, the SS is slightly degraded. The SS of the CLSB-BRFET is much smaller than that of the NBRFET with NiSi S/D electrodes. The average SS of the proposed CLSB-BRFET is about 80 mV/dec, while the minimum SS of the NBRFET with NiSi S/D electrodes is larger than 100 mV/dec. As Fig. 7c shows, the V_TH_ is not obviously affected by V_D_, the V_TH_ of the conventional NBRFET is approximately 0 V, and the V_TH_ of the proposed CLSB-NBRFET is approximately 0.22 eV, which is larger than 0 V; therefore, the proposed device is more suitable for use as a reconfigurable device.

**Fig. 8:**
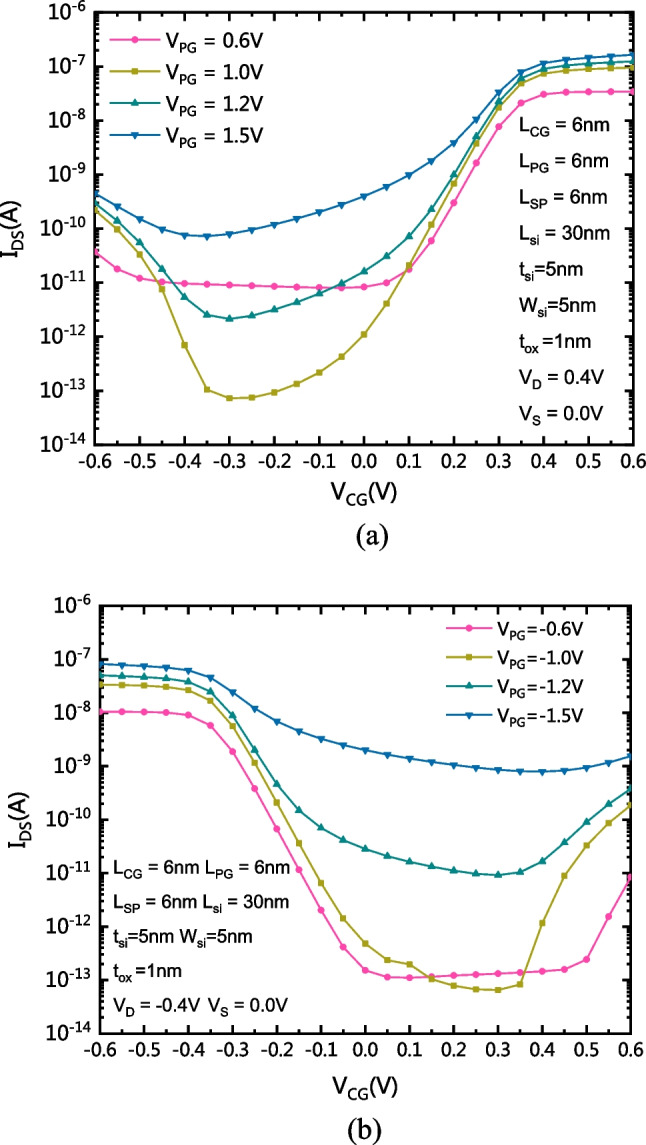
**a** Comparison of the transfer characteristics of the CLSB-BRFET with different V_PG_ in n-mode. **b** Comparison of the transfer characteristics of the CLSB-BRFET with different V_PG_ in p-mode

Figure 8a shows the comparison of the transfer characteristics of the CLSB-BRFET with different V_PG_ in n-mode. Figure 8b shows the comparison of the transfer characteristics of the CLSB-BRFET with different V_PG_ in p-mode. Because two metal materials are used, one of which forms a low Schottky barrier on the interface between the metal and the conduction band and the other forms a low Schottky barrier on the interface between the corresponding metal and the valence band, it is necessary to apply sufficient voltage to the PG to determine the conduction mode. If V_PG_ is too low, it causes the opposite type of carriers to be much easier to pass through and generate a leakage current. The forward current can be improved by increasing V_PG_. However, an excessively large V_PG_ causes an increase in the potential difference between the PG and CG, thus increasing the leakage current. Therefore, the subthreshold characteristics degrade. By considering the influence of V_PG_ on the reverse, static and forward properties, the optimized value of V_PG_ is approximately 1 V in n-mode, while the optimized value of V_PG_ is approximately − 1 V in p-mode.

**Fig. 9:**
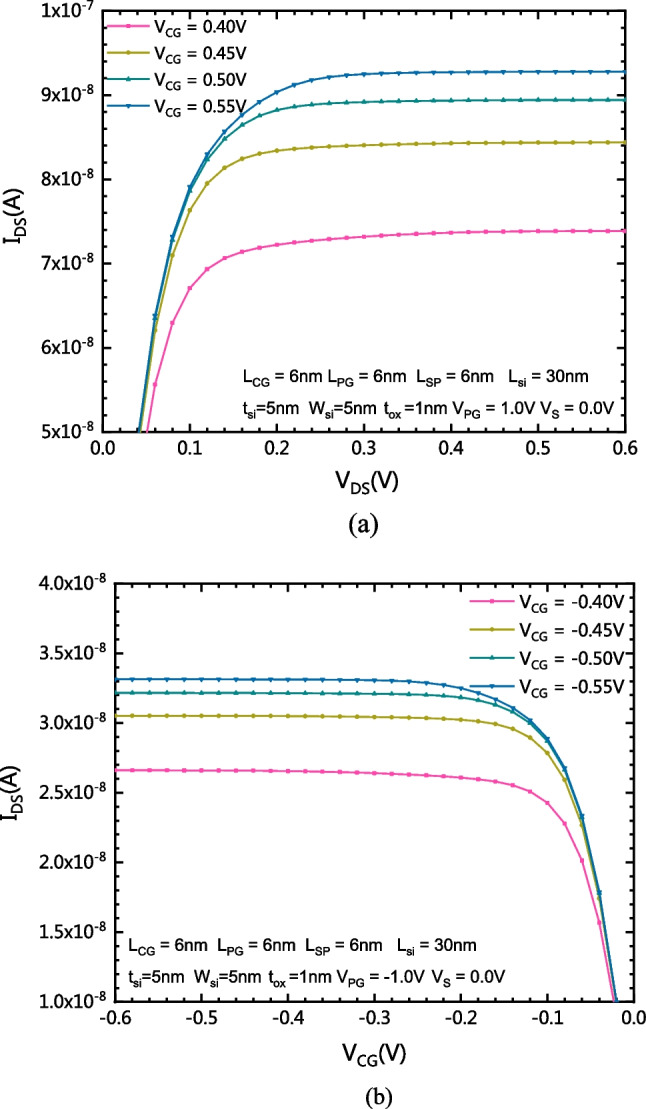
**a** The output characteristics of the proposed CLSB-BRFET with different V_CG_ in n-mode. **b** The output characteristics of the proposed CLSB-BRFET with different V_CG_ in p-mode

Figure 9a shows the output characteristics of the proposed CLSB-BRFET with different V_CG_ in n-mode. Figure 9b shows the output characteristics of the proposed CLSB-BRFET with different V_CG_ in p-mode. The forward-on-state saturation current is strictly restricted by V_CG_. Thereafter, the proposed CLSB-BRFET has a good inhibition effect on the early effect. The output characteristic enters from the linear region into the saturation region as V_DS_ increases. The drain saturation current increases with increasing gate voltage.

**Fig. 10:**
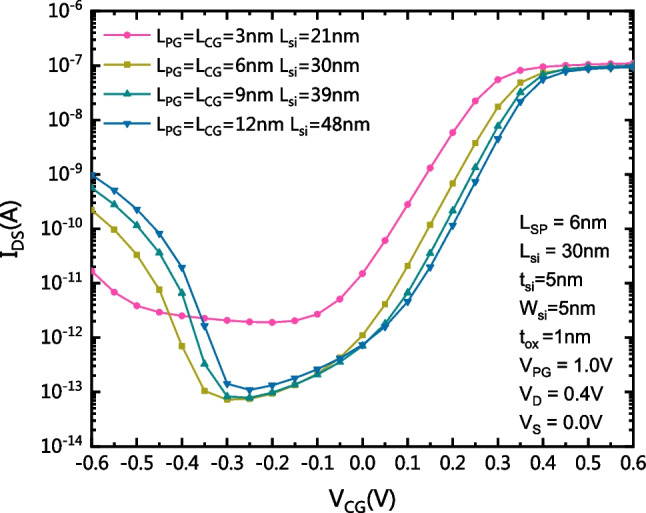
The transfer characteristics of the proposed CLSB-BRFET with different L_CG_ and L_PG_ values

Figure 10 shows the transfer characteristics of the proposed CLSB-BRFET with different L_CG_ and L_PG_. The change in L_CG_ and L_PG_ has no obvious effect on the transfer characteristics unless L_CG_ and L_PG_ are reduced to 3 nm. This is similar to the short channel effect of conventional MOS devices. When L_CG_ and L_PG_ are changed, the curve translates slightly.

**Fig. 11:**
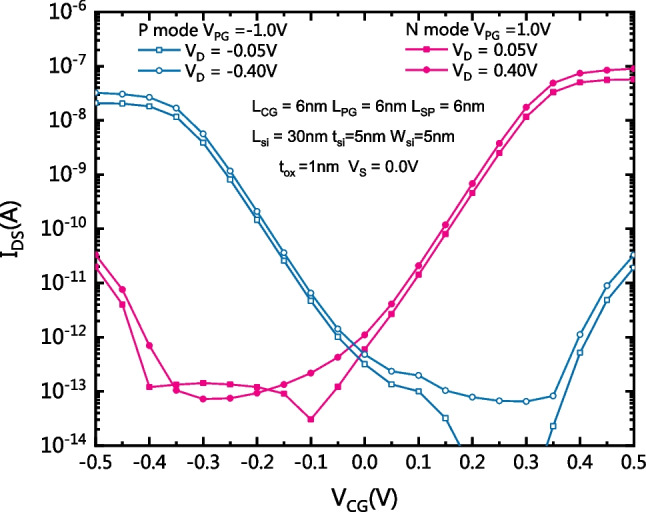
Reconfigurable characteristics of the proposed CLSB-BRFET

Figure 11 shows the reconfigurable characteristics of the CLSB-BRFET. V_PG_ determines the conduction mode. When the PG, the CG and the drain electrode are all positively biased, electrons flow from the ErSi source into the conduction band to form the forward current. The proposed CLSB-BRFET operates in the turn-on state of the n-mode. Similarly, when the PG, CG and the drain electrode are all negatively biased, the holes flow from the PtSi source into the valence band to form the forward current. The proposed CLSB-BRFET operates in the turn-on state of the p-mode. If V_D_ is increased in both modes, the forward on-state current is increased. Moreover, the reverse leakage current is also increased slightly. In both modes, the I_on_/I_off_ ratio is approximately 10^5^.

## Conclusions

In this work, a complementary low-Schottky-barrier source/drain (S/D)-based nanoscale dopingless bidirectional RFET (CLSB-BRFET) is proposed. Two different metal materials are adopted to form two different types of Schottky barriers on the interface between the S/D and silicon. Both a low Schottky barrier for the conduction band and a low Schottky barrier for the valence band are formed simultaneously. Therefore, more carriers from the source electrode can easily flow into the semiconductor region through thermionic emission compared to the conventional BRFET, which generates carriers through the band-to-band tunneling (BTBT) effect. A higher forward conduction current, sharper subthreshold slope and larger I_on_/I_off_ ratio are achieved compared to conventional BRFET operation. If V_PG_ is appropriately selected and large enough, V_D_ does not affect the I_on_/I_off_ ratio. The average SS of the proposed CLSB-BRFET is approximately 100 mV/dec, while the minimum SS of the NBRFET with NiSi S/D electrodes is larger than 100 mV/dec. The optimized value of V_PG_ is approximately 1 V in n-mode, while the optimized value of V_PG_ is approximately − 1 V in p-mode. The change in L_CG_ and L_PG_ has no obvious effect on the transfer characteristics unless L_CG_ and L_PG_ are reduced to 3 nm. V_PG_ determines the conduction mode of the proposed CLSB-BRFET. When the PG, the CG and the drain electrode are all positively biased, electrons flow from the ErSi source into the conduction band to form the forward current. The proposed CLSB-BRFET operates in the turn-on state of the n-mode. Similarly, when the PG, CG and the drain electrode are all negatively biased, the holes flow from the PtSi source into the valence band to form the forward current. The proposed CLSB-BRFET operates in the turn-on state of the p-mode. In both modes, the proposed CLSB-BRFET has good on-state conduction characteristics and low static power consumption, and the I_on_/I_off_ ratio in both n-mode and p-mode reaches approximately 10^5^.

## Data Availability

All available data and material are original work. All data have been clearly provided in the manuscript without additional data and supporting materials. The datasets used and/or analyzed during the current study are also available from the corresponding author on reasonable request.

## Abbreviations

RFET: The reconfigurable field effect transistor
BRFET: Bidirectional RFET
CMOS: Complementary metal oxide semiconductor
PG: Program gate
BTBT: Band-to-band tunneling
MOSFET: Metal oxide semiconductor field effect transistor
SB-MOSFET: Schottky barrier MOSFET
ϕ_b__p_: The height of the Schottky barrier for holes in the valence band
ϕ_b__n_: The height of the Schottky barrier for electrons in the conduction band
S\D: Source/drain
CLSB-BRFET: Complementary low Schottky barrier source/drain (S/D)-based nanoscale dopingless bidirectional RFET
NiSi: Nickel silicide
PtSi: Platinum silicide
ErSi: Erbium silicide
L_si_: The length of the semiconductor (silicon)
L_CG_: The length of the control gate
L_PG_: The length of the program gate
L_sp_: The length of the spacer
t_ox_: The HfO_2_ oxide layer thickness
t_si_: The thickness of the semiconductor (silicon)
W_si_: The width of semiconductor (silicon)
ε_HfO2_: The relative permittivity of HfO_2_
ε_spacer_: The relative permittivity of the spacer
qϕ_bn__1_: The barrier height between the ErSi S/D electrodes and the conduction band of the silicon
qϕ_bp__1_: The barrier height between the ErSi S/D electrodes and the valence band of the silicon
qϕ_bn__2_: The barrier height between the PtSi S/D electrodes and the conduction band of the silicon
qϕ_bp__2_: The barrier height between the PtSi S/D electrodes and the valence band of the silicon
qϕ_bn__0_: The barrier height between the NiSi S/D electrodes and the conduction band of the silicon
qϕ_bp__0_: The barrier height between the NiSi S/D electrodes and the valence band of the silicon
V_DS_: The drain-to-source voltage
V_GS_: The control gate to source voltage
V_PG_: The program gate voltage
E_FMS_: The Fermi energy level of the source electrode
E_FMD_: The Fermi energy level of the source electrode
n: The electron concentration
n_s_: The surface electron concentration
p: The hole concentration
p_s_: The surface hole concentration
E_FN_: The electron quasi-Fermi level
E_FP_: The hole quasi-Fermi level
N_C_: The effective density of states of the conduction band
N_V_: The effective density of states of the conduction band
E_C_: The band energy of the bottom of the conduction band
E_V_: The band energy of the top of the valence band
V_TH_: Threshold voltage
I_on_/I_off_: The on-state and off-state current ratio
SS: The subthreshold swing

## Code availability

Source code is available upon request from the corresponding author.
